# Long-Term Survival of Bifocal Paraganglioma: A Case Report

**DOI:** 10.7759/cureus.59048

**Published:** 2024-04-26

**Authors:** Nourelhouda Mouhib, Fatima Benhjar, Soufiane Berhili, Mohamed Moukhlissi, Loubna Mezouar

**Affiliations:** 1 Radiation Therapy, Mohammed VI University Hospital, Faculty of Medicine and Pharmacy, Mohammed First University, Oujda, MAR; 2 Radiation Oncology, Mohammed VI University Hospital, Faculty of Medicine and Pharmacy, Mohammed First University, Oujda, MAR

**Keywords:** surgery, secreting tumor, head and neck cancer, radiation therapy, bifocal paraganglioma

## Abstract

Paragangliomas are sympathetic and parasympathetic para-ganglia neuroendocrine tumors of the autonomic nervous system.

We analyzed a bifocal paraganglioma case of a 52-year-old patient in December 2013 with hearing loss and right ear pain, headaches, episodes of vomiting, and abdominal pain ten months before her medical consultation. The diagnosis of a right tympano-jugular glomus paraganglioma was based on cerebral magnetic resonance imaging and treated with radiotherapy. In 2016, the patient presented with worsening digestive symptoms; therefore, a second mesocolic localization was suspected by abdominal computed tomography and was histologically confirmed on the resection specimen of the mass. The surgery was the only treatment. After a follow-up of 11 years, the patient remained in good condition.

Paraganliomas are rare tumors, their bifocal location in our patient represents an even rarer entity. Given the nonspecific symptomatology, the diagnosis of the retroperitoneal location simultaneously with that of the head and neck was difficult.

Our objective is to emphasize the staging workup for paraganglioma, although it is mostly a benign tumor with slow growth.

## Introduction

Paragangliomas are neuroendocrine tumors that develop from chromaffin cells of the para-ganglion system; 90% are localized in the adrenal gland (pheochromocytoma), and 10% are extra-adrenal, occurring in the abdomen (85%), the thorax (12%), and less frequently in the head and neck (3%) [1].

 Paragangliomas can be sporadic or multifocal, and may secrete or not secrete catecholamines. Paraganglioma is a rare tumor with slow development and high vascularity which creates diagnostic and therapeutic challenges.

Several factors contribute to morbidity, mortality, and prognosis in patients diagnosed with paragangliomas, among which are catecholamine hypersecretion, histologic parameters, infiltrative growth, incomplete resection, and metastatic disease.

Early detection, complete tumor resection, and appropriate clinical follow-up are key management strategies for patients with paragangliomas.

## Case presentation

A 52-year-old patient with hypertension and non-insulin-dependent diabetes, which had been evolving for a year before her consultation in 2013 was analyzed. Ten months before, the patient manifested symptoms including right ear pain and hypoacusis, tinnitus, headaches, occasional vomiting episodes, and intermittent abdominal pain. The otological examination revealed a bluish mass filling the external auditory canal, and the neurological examination showed signs of incipient facial paralysis. Noradrenaline levels were measured at 6613 ng/l in the blood and 1958 µg/24h in the urine. Vanillylmandelic acid was 25.9 mg/24 h (18.4 µmol/mmol creatinine) (Table 1). 

**Table 1:** Patient values and the corresponding reference values of biological analysis.

Biological analysis	Patient values	Reference values
Noradrenaline (blood)	6613 ng/l	< 507 ng/l
Noradrenaline (urine)	1958 µg/24 h	15-84 µg/24 h
Vanillylmandelic acid	18.4 µmol/mmol creatinine	0.8-2 µmol/mmol creatinine

**Figure 1:**
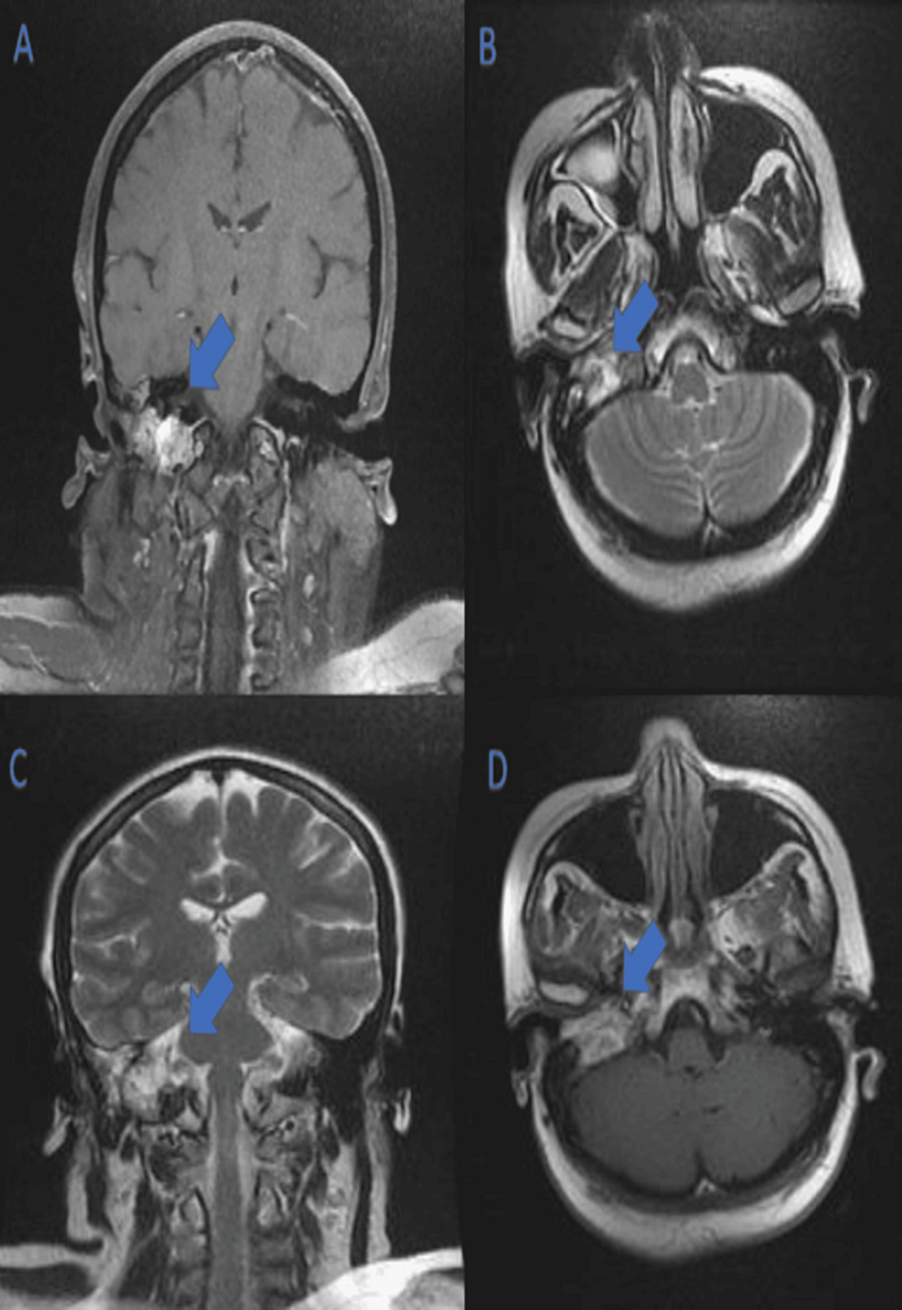
Cerebral MRI with angiographic sequences. (A) Post-contrast T1-weighted coronal section; (B) axial T2 weighted section; (C) coronal T2 section, and (D) post-contrast T1 weighted axial. A-D images show a lesion centered on the jugulo-tympanic axis in hypointense T1 and hyperintense. T2 post-contrast images show intense enhancement of the lesion.

A CT scan of the petrous bone followed by a cerebral MRI with angiographic sequences confirmed the presence of a lytic tumoral process of the right petrous bone and jugular foramen, suggesting a jugulo-tympanic glomus tumor (Figure 1). The cerebral MRI images depicted are as follows: post-contrast T1-weighted coronal section (Figure 1A), axial T2-weighted section (Figure 1B), coronal T2 section (Figure 1C), and post-contrast T1-weighted axial section (Figure 1D). These images show a lesion centered on the jugulo-tympanic axis in hypointense T1 and hyperintense T2. The post-contrast images show intense enhancement of the lesion.

**Figure 2:**
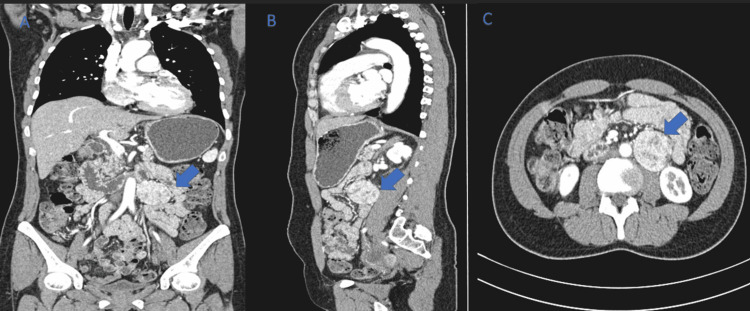
Thoraco-abdominopelvic CT scans. (A) Coronal section; (B) sagittal section, and (C) transverse section of the abdominal CT showing a well-defined heterogeneous hypodense mass in the left para-aortic region above the level aortic bifurcation enhanced after injection of the contrast product. A 42 mm x 41 mm lesion without signs of loco-regional invasion was measured.

An abdominal ultrasound was normal. The patient was treated with 3D conformal radiotherapy targeting the tumor, with a total dose of 54 Gy/30 fractions at 1.8 Gy/fraction by four fields including left lateral, right lateral, left oblique, and right posterior oblique, spread over 48 days. The evolution is marked by the drying up of otological symptoms except for hypoacusis. In 2016, a thoraco-abdominopelvic CT scan was requested due to worsening digestive symptoms like diffuse abdominal pain and vomiting, revealing a left-lateralized retroperitoneal tissue mass measuring 42 mm x 41 mm (Figure 2).

**Figure 3:**
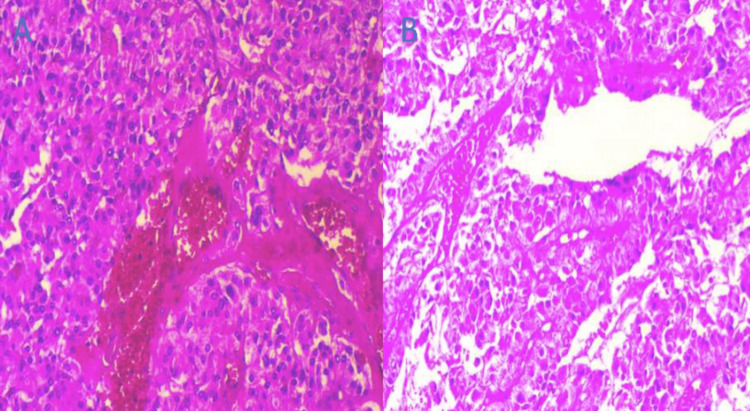
Histological images showing (A) para-aortic paraganglioma, and (B) cellular details and zellballen architecture.

The levels of catecholamines and derivatives were normal. Diagnostic and therapeutique surgery was performed and the anatomopathological study of the surgical specimen confirmed the diagnosis of a mesocolic paraganglioma (Figure 3).

The patient underwent clinical monitoring every three months for two years, then every six months, and radiological monitoring with an annual cerebral MRI. After 11 years of follow-up, the patient remains well-controlled. 

## Discussion

Paragangliomas are rare tumors with an incidence between 2 and 8 per million [2], mostly solitary; and can be multiple in the context of familial tumor syndromes [1]. Our case is sporadic and the tumor of the patient occurred, in accordance with the literature, in the fourth decade of her life at two different locations: right jugulo-tympanic and left retroperitoneal mesocolic [3].

Head and neck paragangliomas account for 0.6% of tumors in this anatomical region [4], with a higher incidence in women, affecting two to three times more females than males [1]. These tumors are typically located around blood vessels and nerves, with the most common sites being the carotid bifurcation, followed by the jugulo-tympanic area, and less frequently, the vagal region. In contrast, retroperitoneal paragangliomas would be less frequent than other abdominal locations, with a slight male predominance [4].

The clinical symptomatology was variable and often nonspecific. It can include signs related to the overproduction of catecholamines: high blood pressure, headaches, palpitations, profuse sweating, vomiting and/or signs related to the tumoral syndrome: tinnitus, hypoacusis, vertigo, dysphonia, dysphagia, or Horner's syndrome due to possible cranial nerve involvement. Other symptoms such as abdominal pain, anxiety, chills, paleness, and digestive disorders are less specific [5].

Paraganglial tumors of the head and neck manifesting as a lytic syndrome rarely release catecholamines and are therefore less vasoactive, unlike abdominal paragangliomas, which secrete in 85% of cases [6]. Nevertheless, some patients can be perfectly asymptomatic. Thus, there is often a diagnostic delay; in fact, the average time between the onset of symptoms and diagnosis is at least three years [7].

The most sensitive and specific biological tests for these tumors are realized through urinary measurements of metanephrine and normetanephrine, and through plasma-free metanephrine levels [5], although these measurements can be normal even for secreting tumors. In brief, measurement of catecholamines and vanillylmandelic acid is less sensitive but commonly practiced [5].

MRI with arterial phase sequences is a preferred imaging modality for paraganglioma in the head and neck region and CT scans are favored for other locations like thorax and abdomen [8].

Magnetic resonance imaging is the most sensitive imaging modality for evaluation of paragangliomas given its superior soft tissue resolution. The presence of intralesional hemorrhage, slow vascular flow, and vascular flow voids “salt and pepper” are more specific for paragangliomas when distinguished from similar-appearing tumors such as schwannomas, meningiomas, endolymphatic sac tumors, myeloma, and metastasis. This appearance is absent if the tumor is smaller than 1 cm [8].

On computed tomography, paragangliomas most commonly appear homogenous, with avid enhancement after administration of intravenous contrast. CT imaging is excellent for the evaluation of osseous involvement [8].

Our patient presented various symptoms and signs, and the diagnosis of a jugulo-tympanic paraganglioma was based on cerebral magnetic resonance imaging with angiographic sequences. The biological analysis of noradrenaline and vanillylmandelic acid was positive, classifying the tumor as one of the rare-secreting paragangliomas of the head and neck.

The cerebral MRI of our patient was typical and showed a tissue process centered on the rock and the right jugular golf course poorly limited in iso-signal T1; heterogeneous hypersignal T2 with intense enhancement after contrast. CT of the abdominal level showed a rounded left latero-aortic retroperitoneal tissue mass with regular contours enhanced, in a heterogeneous manner suggesting a paraganglioma, a leiomyosarcoma, and a gastrointestinal stromal tumor.

An assessment of tumor extension is warranted due to the risk of metastasis to non-chromaffin tissues and lymph nodes, which ranges from 10% to 17% [9] and is slightly lower for others at 5% to 13.5% [10]. This metastatic potential represents the primary criterion for malignancy in paragangliomas and given the probability of having multiple masses concurrently. Conventional imaging by CT or MRI is used depending on the location [9,11]. 

Currently, functional imaging is included in the extent of disease evaluation of a paraganglioma [11].

In the case of our patient, after the diagnosis of tympano jugular paraganglioma, investigations looking for metastases or other locations were missing. Our consideration of bifocality was based on the evolving clinical symptoms in favor of catecholamine hypersecretion, although biological tests were normal in 2016.

A biopsy of a suspected or diagnosed paraganglioma is to be avoided, as it carries a risk of hemorrhage [12].

Histologically, resected specimens of paragangliomas are often well-circumscribed lesions, with tumors generally displaying a classic architecture in the form of nests of cells with an alveolar pattern (traditionally called "zellballen") against a well-vascularized stroma. The nuclei of tumor cells show vesicular chromatin and visible nucleoli, and the cytoplasm is often granular and intensely basophilic. Immunohistochemically, tumors invariably express neuroendocrine markers such as chromogranin A and synaptophysin [1].

The management of paragangliomas requires a multidisciplinary approach. Surgery is the only curative treatment provided. It achieves survival rates of 75% and 45% at 5 and 10 years respectively. Due to the high vascularity of these tumors, some authors recommend preoperative embolization of the tumor [5].

Radiotherapy is a therapeutic alternative reserved for post-surgical recurrences and non-resectable tumors, providing local tumor control without significant morbidity. Local recurrence or degeneration is possible, as described in the literature [11].

The therapeutic decision of the multidisciplinary consultation meeting regarding the jugulo-tympanic paraganglioma of our patient was external 3D conformal radiotherapy with a total dose of 54 Gy/30 fractions (1.8 Gy/fraction) scheduled over a 49-day period. For the retroperitoneal location, excision surgery by laparotomy was performed.

Long-term follow-up is recommended by the clinic: blood pressure monitoring, annual or biennial MRI. The risk of recurrence depends on young age, tumor size, and hereditary form [7].

Our patient, after eleven years since the diagnosis of jugulo-tympanic paraganglioma and eight years postoperative for retroperitoneal paraganglioma, remains clinically stable, still followed for hypertension; she retains right hypoacusis, with no recurrence on imaging.

## Conclusions

Paragangliomas are rare neuroendocrine tumors developed from the paraganglionic nervous system, which is distributed around the vessels from the base of the skull to the pelvis in single or multiple locations and are most often observed in young adults around 40 years old.

Imaging is fundamental not only for the diagnosis of paragangliomas but also for the extension assessment, which should not be neglected. Even though paragangliomas generally have a benign behavior, they can recur or metastasize, especially those located in the head and neck or in multiple locations.

Radiotherapy is a therapeutic alternative for paragangliomas where surgery is associated with high morbidity. Our patient remains in good control after a follow-up of 11 years, indicating the benign nature of tumor.
